# Prostaglandin D_2_ metabolites as a biomarker of *in vivo* mast cell activation in systemic mastocytosis and rheumatoid arthritis

**DOI:** 10.1002/iid3.94

**Published:** 2015-11-25

**Authors:** Catherine Cho, Anna Nguyen, Katherine J. Bryant, Sean G. O'Neill, H. Patrick McNeil

**Affiliations:** ^1^ Faculty of MedicineUniversity of New South WalesSydney 2052Australia; ^2^ Faculty of Medicine and Health SciencesMacquarie UniversitySydney 2109Australia

**Keywords:** Biomarker, mast cell, prostaglandin D metabolites, rheumatoid arthritis, systemic mastocytosis, tetranor PGDM

## Abstract

Mast cells (MCs) participate in diseases such as systemic mastocytosis (SM) and allergic conditions. Less well understood is the role of MCs in non‐allergic inflammatory disorders like rheumatoid arthritis (RA). Studying definitive roles for MCs in human diseases has been hampered by the lack of a well‐accepted biomarker for monitoring *in vivo* MC activation. This study aimed to investigate the utility of urinary tetranor PGDM (T‐PGDM) as a biomarker of *in vivo* MC activation in patients with SM, and apply this biomarker to assess MC involvement in relation to RA disease activity. A prospective, cross‐sectional cohort study was conducted to measure a major urinary metabolite of prostaglandin D_2_, T‐PGDM. Urine samples were collected from patients with RA (*n *= 60), SM (*n *= 17) and healthy normal controls (*n *= 16) and T‐PGDM excretion was determined by enzyme immunoassay as nanograms per milligram of urinary creatinine (ng/mg Cr). Mean urinary T‐PGDM excretion was significantly higher (*p* < 0.01) in patients with SM compared to controls (37.2 vs. 11.5 ng/mg Cr) with 65% of SM patients showing elevated levels. One third of patients with RA had elevated T‐PGDM excretion, and the mean level in the RA group (20.0 ng/mg Cr) was significantly higher than controls (*p* < 0.01). Medications inhibiting cyclooxygenase reduced T‐PGDM excretion. Urinary T‐PGDM excretion appears promising as a biomarker of *in vivo* MC activity and elevated levels in 33% of patients with RA provides evidence of MC activation in this disease.

## Introduction

Mast cells (MCs) are granular, inflammatory cells with major roles in the immune and tissue repair systems 1. Many beneficial immunological and physiological functions of MCs occur by their release of a range of mediators including histamine, the MC‐specific serine proteases, tryptase and chymase, lipid‐derived mediators including leukotrienes and prostaglandin D_2_ (PGD_2_), and a broad spectrum of cytokines 2, 3. Excessive or inappropriate mediator release is responsible for the clinical manifestations seen in pathological conditions in which MCs play a primary role. However, an important difficulty with studying MC involvement in human disease is the lack of a universally accepted sensitive biomarker that accurately reflects *in vivo* MC activity.

Measuring the quantity of mediators released from MCs provides a pathway to assessing MC activity *in vivo*. Indeed, measurements of metabolites including tryptase and histamine in both serum and urine are employed as diagnostic tools in mastocytosis syndromes 4. Additionally, these metabolites have been analysed in anaphylaxis and asthma. Existing data on metabolite concentrations in such patients, however, has not been consistent, with varying reports of sensitivity and specificity 5, 6. Consequently, there has been interest in other MC metabolites including PGD_2_ as potential biomarkers for *in vivo* activity 7. PGD_2_ is produced in MCs from arachidonic acid derivatives by cyclooxygenase and haematopoietic PGD‐synthase 8. Whilst evidence suggests PGD_2_ production occurs in platelets, macrophages, T helper cells and dendritic cells, levels are 100–1000 times lower than that synthesised by activated MCs indicating that PGD_2_ production largely reflects MC activity 9. *In vivo* and *in vitro*, PGD_2_ is an unstable compound and is rapidly degraded into D‐, F‐ and J‐ring metabolites, which are excreted as more stable urinary metabolites 7. In a study by Liston and Roberts, 25 predominantly F‐ring metabolites were isolated from a healthy male volunteer, of which the F‐ring metabolite 9α,11β‐dihydroxy‐15‐oxo‐2,3,18,19‐tetranorprost‐5‐ene‐1,20‐dioic acid was found to be abundant 10. More recently, the D‐ring metabolite 11,15‐dioxo‐9α‐hydrox‐2,3,4,5‐tetranor‐prostan‐1,20‐dioic acid [tetranor PGDM (T‐PGDM)] was discovered by Song et al. and shown to be more abundant than the F‐ring metabolites 7, 11.

Elevated levels of MC‐derived mediator metabolites are seen in systemic mastocytosis (SM), a disease that results from the clonal proliferation of abnormal MCs in one or more extracutaneous organs 12. Existing biomarkers, histamine and tryptase have been shown to be elevated in this disease, reflecting increased MC load and activity 6. Elevated PGD_2_ metabolite levels have also been documented in SM patients 13. The F‐ring PGD_2_ metabolite, 9α,11β‐PGF_2_ and D‐ring T‐PGDM have been found to increase during an exacerbation of anaphylaxis and aspirin‐induced asthma, returning to baseline during resolution 9, 11, 14, 15. As MCs are the major source of PGD_2_ and exacerbation of anaphylaxis and asthma are accompanied by IgE‐mediated MC activation and subsequent elevated prostanoid production, measuring urinary T‐PGDM excretion may be a useful strategy to assess *in vivo* MC activity.

There is a growing body of evidence to suggest MCs play a far greater role in human disease than is currently understood 16. Rheumatoid arthritis (RA), an autoimmune disease manifest clinically by chronic inflammation of joints, is one of many diseases with documented MC involvement 17. Rheumatoid synovium is heavily infiltrated with inflammatory cells, mainly lymphocytes and macrophages. Less appreciated are the expansions in MC density and subtype distribution 18, 19. These results are supported with data from mouse studies which demonstrate resistance to RA models in MC‐deficient mice and attenuated joint inflammation in mice with reduced MC load 20. Additionally, degranulation, the hallmark of MC activation, has been observed in a greater proportion of MCs in RA affected joints in comparison to MCs in normal joints (10–15% vs. <1%) 21, likely by non‐IgE‐mediated pathways, given that human synovial MCs are readily activated by IgG‐containing immune complexes via FcγRI 22. Monitoring MC activity using a biomarker would assist with current understanding of MC involvement in RA.

## Methods

### Subjects and study design

A prospective, cross‐sectional cohort study was undertaken to collect spot urine samples from 93 subjects, consisting of RA patients (*n *= 60), SM patients (*n *= 17) and healthy controls (*n *= 16), the latter of whom had no clinical history of inflammatory or autoimmune condition requiring medication. Diagnoses of RA 23 and SM 24 were made by their specialist clinicians based on clinical manifestations and investigation findings. Permission to conduct the study was obtained from the Human Research Ethics Committee of South Western Sydney Local Health District and all participants gave written informed consent.

### Sample collection and analysis and clinical evaluation

Participants provided a urine sample into a sterile container, and then samples were stored in aliquots at −80°C until analysis. Participant characteristics are outlined in Table 1. Patients with RA were clinically assessed for the number of tender or swollen joints at the time of specimen collection to determine their Disease Activity Score in 28 joints (DAS28) and the results of serum levels of C‐reactive protein (CRP) taken as part of routine assessment at the time of review recorded. Combined with a patient global assessment of activity, a CRP‐DAS28 score was calculated for each patient as described 25.

**Table 1:** Demographic and clinical characteristics of participants.

Characteristic	Controls (*n* = 16)	RA (*n* = 60)	SM (*n* = 17)
Age in years (range)	44.8 (21–78)	57.9 (22–88)	51.5 (29–69)
Gender (F:M)	2.2:1	2.3:1	4.7:1
Mean CRP‐DAS28	–	3.14	–
Mean CRP (mg/L)	–	7.95	–

T‐PGDM concentration in urine samples was determined using an enzyme immunoassay (EIA) kit (Cayman Chemical Company, Ann Arbor, MI), as per the manufacturer's instructions. The concentration of T‐PGDM in each sample was calculated from the standard curve and corrected for initial dilution. Creatinine concentration in parallel urine aliquots was determined using spectrophotometry, and T‐PGDM excretion was calculated in nanograms per milligram of urinary creatinine (ng/mg Cr).

### Statistical analysis

T‐PGDM excretion values in the 93 participants (range 3.1–87.1 ng/mg Cr, *n *= 93) were log‐transformed to normalise the data. An upper limit for normal urinary T‐PGDM excretion was calculated from the mean plus interquartile range of the log‐transformed values of healthy controls. Analysis of urinary T‐PGDM excretion between participant groups was performed using one way ANOVA with Tukey correction. The effects of medications on T‐PGDM excretion was analysed using a one‐tailed *t*‐test. Pearson's correlation was used to examine parameters of disease activity and urinary T‐PGDM excretion in RA patients. Geometric means ± standard deviation of T‐PGDM excretion between groups were calculated by back‐transformation of the log‐transformed data. *p* values <0.05 were considered as statistically significant.

## Results

### Demographic and clinical characteristics of participants

The ages of participants in the three groups ranged from 21 to 88 years (Table 1). The healthy control group were significantly younger compared to patients with RA (*p *< 0.01), but was not statistically different to patients with SM. The RA and SM groups were statistically similar with respect to age.

Patients with RA had variable disease activity (CRP‐DAS28 ranged from 0.97 to 6.02), and the mean CRP‐DAS28 of the overall group was 3.14. Nearly half (*n *= 26) of the patients with RA had moderate disease activity (CRP‐DAS28 = 3.2–5.1) and 22 were in remission (CRP‐DAS28 < 2.59). Of the remaining 12 patients with RA, 3 had high and 9 had mild disease activity, respectively 25. Most patients with RA were being actively treated with synthetic or biological disease modifying anti‐rheumatic drugs (DMARDs), most commonly combinations of methotrexate and/or leflunomide with an anti‐TNF agent, anti‐IL‐6‐receptor blocker, or other biological DMARD. In addition, 27 patients with RA were taking the oral corticosteroid, prednisone in doses ranging from 2 to 15 mg daily with a mean daily dose of 6.6 mg. Only six patients were taking non‐steroidal anti‐inflammatory drugs (NSAIDs), which in four cases consisted only of low‐dose aspirin (mean 116 mg/day).

All patients with SM were clinically stable but were taking at least one medication, with many patients using multiple medications. Thirteen were using anti‐H1 and anti‐H2 histamine receptor blockers, and in addition four patients were taking the MC stabilising drugs, cromoglycate and/or ketotifen. Two patients were using monthly omalizumab, and three patients were prescribed NSAIDs.

### T‐PGDM excretion in healthy control, SM and RA groups

**Figure 1:**
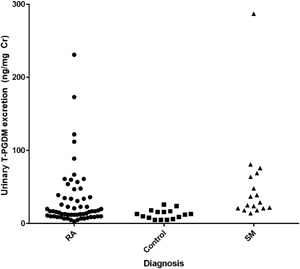
Urinary T‐PGDM excretion in patients with rheumatoid arthritis (RA), systemic mastocytosis (SM) and healthy controls.

Mean urinary T‐PGDM excretion in healthy controls was 11.5 ± 1.7 ng/mg Cr with a range from 5.0 to 26.1. T‐PGDM excretion levels >24.0 ng/mg Cr were considered elevated based upon a cut‐off defined by the mean plus the interquartile range of log‐transformed values. Compared to healthy controls, the mean urinary T‐PGDM excretion was significantly elevated in the group of patients with SM (37.2 ± 2.1 ng/mg Cr; *p* < 0.01), with 11 of 17 patients (65%) having levels above the upper limit of normal (Fig. 1).

In the *n *= 60 group of patients with RA, the mean T‐PGDM excretion was 20.0 ± 2.5 ng/mg Cr, which was elevated compared to healthy controls (*p* < 0.01). Furthermore, 20 of the 60 patients with RA (33%) were found to have levels of urinary T‐PGDM excretion above the normal range measured in healthy controls.

### T‐PGDM excretion in RA and disease activity

There was no significant correlation between T‐PGDM excretion levels and the calculated CRP‐DAS28 scores (*p *= 0.545, *r *= 0.080). Additionally, no significant correlation was detected between T‐PGDM and the individual DAS28 components, number of swollen joints, number of tender joints or patient global assessment (*p *= 0.854, *r *= 0.024; *p *= 0.586, *r *= −0.072; *p *= 0.929, *r *= −0.012). However, a weak correlation between CRP and T‐PGDM excretion was observed (*r *= 0.349), although the strength of this observation is uncertain.

### The effect of medications on T‐PGDM excretion

The effect of drugs that can potentially reduce PGD_2_ production by inhibiting cyclooxygenase was examined by comparing the mean T‐PGDM excretion in patients taking NSAIDs or prednisone with patients not taking these medications (Table 2). Patients taking NSAIDs, whether with RA (*n *= 6), or with SM (*n *= 3) had lower T‐PGDM excretion than those who were not (*n *= 54 and 14, respectively), however the differences did not reach statistical significance (20.4 vs. 33.5 ng/mg Cr, *p *= 0.075 for RA patients and 26.3 vs. 58.2 ng/mg Cr, *p*= 0.064 for SM patients). When the two patient groups were combined, NSAID‐taking patients (*n *= 9) had lower mean T‐PGDM excretion than the *n *= 68 patients not consuming NSAIDs (22.4 vs. 38.6 ng/mg Cr, *p *= 0.015).

**Table 2:** Mean levels of urinary T‐PGDM excretion (ng/mg Cr) in patients with RA and SM in the presence or absence of prednisone and/or NSAIDs.

Treatment Status	Present	Absent	*p*‐value
Patients with RA (*n* = 60)			
Prednisone	22.6 (*n* = 27)	40.1 (*n* = 33)	0.049
NSAID	20.4 (*n* = 6)	33.5 (*n* = 54)	0.075
Both	17.7 (*n* = 4)	33.3 (*n* = 56)	0.074
Either	22.8 (*n* = 29)	41.1 (*n* = 31)	0.042
Patients with SM (*n* = 17)			
NSAID	26.3 (*n* = 3)	58.2 (*n* = 14)	0.064
Patients with either RA or SM (*n* = 77)			
NSAID	22.4 (*n* = 9)	38.6 (*n* = 68)	0.015

Nearly half (*n *= 27) of the 60 patients with RA were taking low doses of prednisone at a mean daily dose of 6.6 mg/day. Decreased urinary T‐PGDM excretion was observed in these patients taking prednisone compared to the 33 patients with RA who were not (22.6 vs. 40.1 ng/mg Cr, *p *= 0.049). Similarly, the mean T‐PGDM excretion was significantly less in RA patients taking either a NSAID or prednisone compared to patients not taking these medications (22.8 vs. 41.1 ng/mg Cr, *p *= 0.042). The lowest mean T‐PGDM excretion levels were observed in the small number (*n *= 4) of patients with RA taking both NSAIDs and prednisone (Table 2).

## Discussion

This study has demonstrated that urinary 11,15‐dioxo‐9α‐hydrox‐2,3,4,5‐tetranor‐prostan‐1,20‐dioic acid (T‐PGDM) is a potentially useful biomarker of *in vivo* MC activity. We observed significantly higher mean urinary T‐PGDM excretion in SM patients compared to healthy controls, with elevated levels of T‐PGDM excretion in a large proportion (65%) of the patients with SM. T‐PGDM excretion levels from the remaining six patients with SM that were below the normal range cut‐off fell between 13.6 and 21.3 ng/mg Cr, all in the top two quartiles of normal values. Our results provide the first data on urinary T‐PGDM excretion in SM patients and is consistent with existing data which demonstrates significantly elevated levels of F‐ring PGD_2_ metabolites in the urine of mastocytosis patients compared to healthy controls 26, 27. While PGD_2_ production has been demonstrated in several other inflammatory cells, elevated urinary T‐PGDM levels in SM is highly supportive of MCs as the primary source of PGD_2_ and subsequently its metabolites 9. Further support for PGD_2_ metabolites arising from *in vivo* MC activation is the consistent finding of significantly elevated PGD_2_ metabolite excretion in other MC associated conditions such as MC activation syndrome, anaphylaxis and asthma following provocation eliciting MC activation 9, 14, 15, 28, 29.

Second, our data has demonstrated through the observation of elevated urinary T‐PGDM excretion in 33% of patients with RA, that MCs are hyperactivated in some patients with this autoimmune disease, supporting current literature that implicates MC involvement in its pathophysiology. A potential caveat is the significantly younger mean age of healthy controls compared to patients with RA and future studies should examine T‐PGDM excretion in a larger control group. Our findings in the RA group corroborate previous studies demonstrating elevated levels of MC‐derived inflammatory mediators in synovial fluid from RA affected joints compared to normal joints 30. Several studies have shown an association between increased synovial MC numbers and disease severity 18, 31. A positive correlation between the composite CRP‐DAS28 score, a validated method of assessing disease activity in RA patients, could be expected based on this observation. However, our results showed only a trend for a correlation between CRP levels and urinary T‐PGDM excretion in our RA cohort. It is noteworthy that CRP levels are the only objective component of the DAS28 score, and the lack of association with more subjective measures such as joint counts and patient global assessments could be due to lack of specificity of these clinical components. Furthermore, MCs may play a bigger role in joint damage rather than inflammation in RA 32, and future studies should study relationships between urinary T‐PGDM excretion and more robust assessments of joint damage in RA such as the Sharp/van der Heijde‐Sharp score 33.

A third important conclusion is that medications that inhibit PGD_2_ production, such as NSAIDs and corticosteroids, lead to reduced PGD_2_ metabolites being excreted in the urine, and thus urinary T‐PGDM excretion levels would tend to under‐estimate the degree of MC activation in patients taking these medications. Song et al. demonstrated that a dose of aspirin at 325 mg reduced urinary T‐PGDM excretion 7. Furthermore, a dose of aspirin from 160 to 1000 mg/day in SM patients can be used to reduce the severity of attacks associated with PGD_2_ release, whilst reducing urinary F‐ring metabolite 9α,11β‐PGF_2_ to normal levels 34.

In summary, the ability to quantitate *in vivo* MC activity through the use of a biomarker can improve our understanding of the role of MCs in human disease and open potential pathways for MC targeted therapies. Urinary T‐PGDM shows promise as a novel biomarker; the non‐invasive nature of measurement of *in vivo* MC activation provides a valuable rationale for use in clinical situations to expand understanding of the role of MCs in diseases, such as RA. However, greater understanding of its kinetics, optimal methods of measurement and impact of medications on *in vivo* PGD_2_ synthesis is necessary.
